# Determination of Pentacyclic Triterpenoids in Plant Biomass by Porous Graphitic Carbon Liquid Chromatography—Tandem Mass Spectrometry

**DOI:** 10.3390/molecules28093945

**Published:** 2023-05-07

**Authors:** Ilya S. Voronov, Danil I. Falev, Anna V. Faleva, Nikolay V. Ul’yanovskii, Dmitry S. Kosyakov

**Affiliations:** Laboratory of Natural Compounds Chemistry and Bioanalytics, Core Facility Center “Arktika”, M.V. Lomonosov Northern (Arctic) Federal University, Northern Dvina Emb. 17, 163002 Arkhangelsk, Russian.ulyanovsky@narfu.ru (N.V.U.)

**Keywords:** pentacyclic triterpenoids, high performance liquid chromatography, HPLC-MS, plant biomass, porous graphitic carbon, hypercarb

## Abstract

Pentacyclic triterpenoids (PCTs), which possess a number of bioactive properties, are considered one of the most important classes of secondary plant metabolites. Their chromatographic determination in plant biomass is complicated by the need to separate a large number of structurally similar compounds belonging to several classes that differ greatly in polarity (monools, diols, and triterpenic acids). This study proposes a rapid, sensitive, and low-cost method for the simultaneous quantification of ten PCTs (3*β*-taraxerol, lupeol, *β*-amyrin, *α*-amyrin, betulin, erythrodiol, uvaol, betulinic, oleanolic, and ursolic acids) by high-performance liquid chromatography-tandem mass spectrometry (HPLC-MS/MS) using porous graphitic carbon (Hypercarb) as a stationary phase capable of hydrophobic retention and specific interactions with analytes. Revealing the effects of the mobile phase composition, pH, ionic strength, and column temperature on retention and selection of chromatographic conditions on this basis allowed for the effective separation of all target analytes within 8 min in gradient elution mode and attaining limits of detection in the range of 4–10^4^ µg L^−1^. The developed method was fully validated and successfully tested in the determination of PCTs in common haircap (*Polytrichum commune*) and prairie sphagnum (*Sphagnum palustre*) mosses, and fireweed (*Chamaenerion angustifolium*) stems and leaves.

## 1. Introduction

Pentacyclic triterpenoids (PCTs) are one of the most important classes of secondary plant metabolites. Being composed of six isoprene units, their backbone consists of 30 carbon atoms and involves five 5- and/or 6-membered rings [1,2,3]. Depending on their structure, PCTs are classified into the lupane, ursane, oleanane, friedelane, serratane, and taraxerane types according to the names of the corresponding parent hydrocarbons; the first three types are most widespread in nature. The most common substituents are one or more hydroxyl groups, a carboxyl group (triterpenic acids), and an oxygen atom in the ketone group. The main dietary source of PCTs is the peels of fruits and berries [1,2]. On an industrial scale, PCTs can also be obtained from the bark of woody plants—an example is birch bark, which contains up to 30% of betulin and some other compounds of this class [3,4].

PCTs exhibit a number of bioactive properties, including anti-HIV, antitumor, antimicrobial, antidiabetic, anti-inflammatory, and antioxidant [5,6,7,8,9,10,11,12,13]. This makes them a promising raw material for obtaining a variety of food additives, pharmaceuticals, and cosmetic products [14,15]. The rapidly growing interest in the practical applications of PCTs necessitates the development of highly sensitive, selective, and rapid methods for their determination in plant biomass and related products.

While the tasks of rapid group screening of PCTs in plant tissues and extracts can be solved by Fourier-transform infrared spectroscopy (FTIR) [16] and matrix-assisted laser desorption/ionization (MALDI) mass spectrometry [17], their detailed analysis is impossible without chromatographic separation. Due to the non-volatility of PCTs, the use of gas chromatography is limited by the need for the derivatization of analytes, which in turn requires additional preliminary purification of the studied extracts [18,19]. In this regard, high-performance liquid chromatography, which allows for the avoidance of tedious and time-consuming sample preparation procedures and the carrying out of direct analyses of plant extracts, can be considered the most promising separation method [20,21,22,23]. Among the detection techniques, atmospheric pressure chemical ionization tandem mass spectrometry (APCI-MS/MS) [18] in the multiple reaction monitoring (MRM) mode demonstrated undoubted advantages, providing the highest selectivity and sensitivity of the analysis [24,25].

The main issue in the HPLC-MS analysis of PCTs is the need for the chromatographic separation of a large number of structurally similar compounds belonging to several classes that differ greatly in polarity and chemical nature (monools, diols, and triterpenic acids). In the case of the most common reversed-phase (RP) chromatography, this results in a long analysis time even in a gradient elution mode and incomplete separation of some compounds, including positional isomers that cannot be distinguished by MS detection, for example, *α*- and *β*-amyrins [2,4]. To overcome this problem, stationary phases with a mixed retention mechanism can be successfully used. Thus, in our previous work [26], ten PCTs, including triterpenic acids, were separated within seven min on an Acclaim Mixed-Mode WAX-1 column, which combines hydrophobic, hydrophilic, and weak anion-exchange (AE) properties. Depending on the mobile phase composition, the retention of analytes can be switched between RP-AE and hydrophilic interactions chromatography (HILIC)-AE regimes, while the contribution of ion-exchange interactions, which play a key role in triterpenic acid separation, can be controlled by changing the pH and ionic strength of the eluent. The variation of several parameters that affect retention allowed for the fine tuning of system selectivity and the fast separation of the most complex analyte mixtures. A similar situation was observed in the separation of PCTs on non-endcapped octadecyl silica stationary phases in supercritical fluid chromatography (SFC). Varying the methanol concentration in the supercritical carbon dioxide led to a significant change in the contributions of hydrophobic and polar (with the residual silanol groups) interactions, with the possibility of a transition from reversed-phase separation of PCTs to the normal phase one, and vice versa [27]. This approach also provided the separation of ten PCTs with the same duration of the analysis as in the case of an HPLC on an Acclaim Mixed-Mode WAX-1 stationary phase. However, due to the absence of ion-exchange interactions with analytes, the stationary phase used SFC did not allow for the baseline separation of triterpenic acids (betulinic, oleanolic, and ursolic), which are characterized by poor retention on octadecyl silica. Despite the high efficiency of PCT separation, both described mixed-mode chromatography techniques have certain disadvantages. The first technique is based on the use of rather expensive chromatographic columns with ion-exchange functionality and is characterized by the complexity of the method optimization, while the second one requires the use of hardly available SFC instrumentation.

The present study is based on the idea that an efficient separation of PCTs can be achieved on an alternative type of stationary phase with a mixed retention mechanism—porous graphitic carbon (PGC). Combining a hydrophobicity similar to RP stationary phases and the capability of retaining polar analytes and even ionic species due to the so-called polar retention (through the charge induction on the polarizable graphite surface) [28,29], PGC was successfully used in the chromatographic analyses of various natural compounds [30,31,32]. However, the available literature on its application to the PCTs separation is scarce and limited to a few papers. Among them, the work of Rhourri-Frih et al. [33], devoted to the determination of the widest range of PCTs (10 analytes of different classes) in natural resinous materials by HPLC-MS with atmospheric pressure photoionization, deserves the most attention. Complete separation was achieved within 50–60 min on a Hypercarb column using an acetonitrile-isopropanol mixture as a mobile phase, which is unacceptable for high-throughput routine analysis.

**Figure 1:**
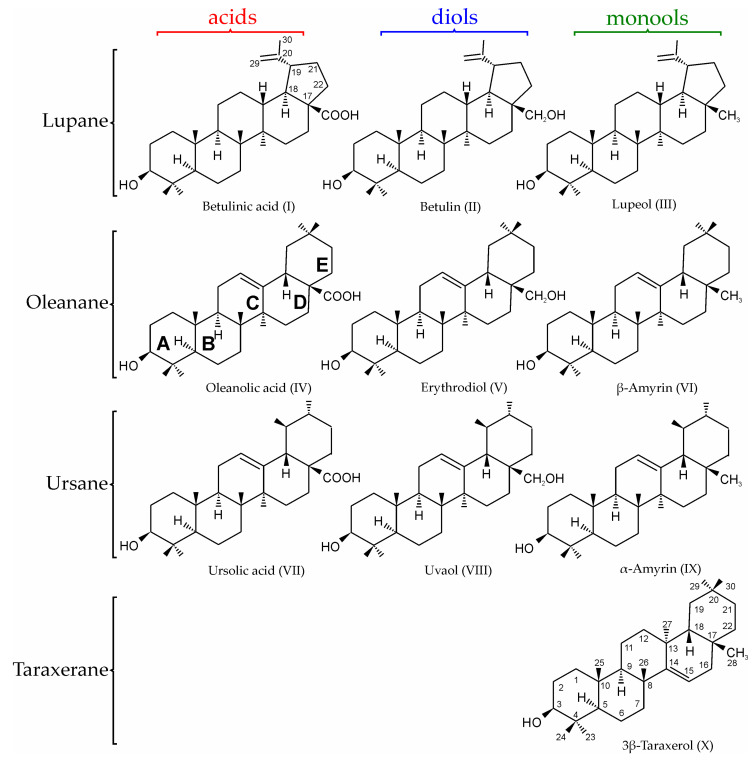
Structural formulas and classification of the studied pentacyclic triterpenoids.

Thus, the aim of our work was to develop and validate a rapid and sensitive method for the quantification of pentacyclic triterpenoids in plant extracts by porous graphitic carbon liquid chromatography—tandem mass spectrometry. Ten compounds (Figure 1) of different classes that are most often found in plant biomass from the boreal zone were chosen as target analytes. They include four monools (3*β*-taraxerol, lupeol, *α*-, and *β*-amyrins), three diols (betulin, erythrodiol, and uvaol), and three triterpenic acids (betulinic, oleanolic, and ursolic).

## 2. Results and Discussion

### 2.1. Retention of PCTs and Selection of the Chromatographic Conditions

**Figure 2:**
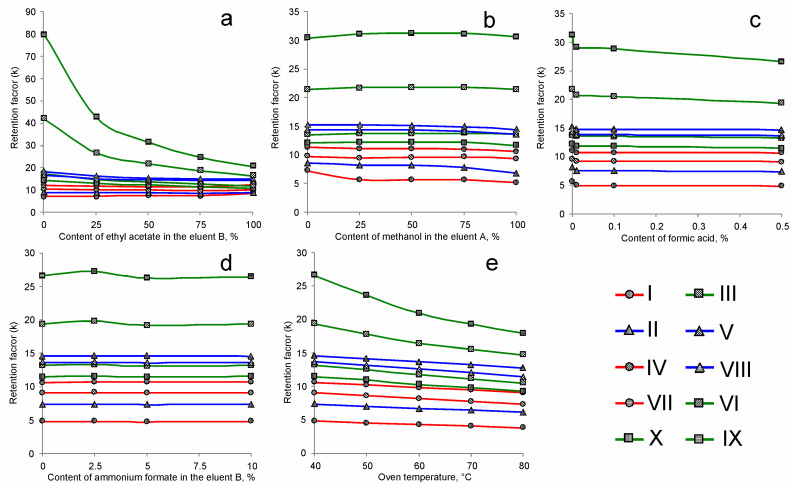
Effect of various factors on the retention of PCTs on the Hypercarb stationary phase (**a**) eluent A: 100% acetonitrile; (**b**) eluent B: 50% of ethyl acetate in isopropanol; (**c**) eluent A: 50% of methanol in acetonitrile and eluent B: 50% of ethyl acetate in isopropanol; (**d**,**e**) eluent A: 50% of methanol in acetonitrile and eluent B: 50% of ethyl acetate in isopropanol, 0.5% of formic acid.

Since PGC (Hypercarb) is characterized by a strong retention of many weakly polar compounds, including PCTs, to ensure the rapidity of the analysis, an approach based on the use of the shortest possible (30 mm) chromatographic column in combination with non-aqueous mobile phases with a high elution power was chosen for the method development. Taking into account the possible mechanisms of analyte retention, the establishment of the chromatographic separation conditions involved studying the effects of the following factors on the retention of PCTs: (i) the mobile phase composition; (ii) the mobile phase pH (formic acid content) and ionic strength (ammonium formate concentration); and (iii) the column temperature (Figure 2).

Preliminary experiments have shown that the rapid separation of PCTs differing greatly in polarity (as monools and triterpenic acids) cannot be achieved in the isocratic elution mode. In this regard, to provide the widest possibilities for tuning the mobile phase elution power, two solvent systems, both consisting of protic and aprotic components, were chosen as eluents in the gradient elution: methanol—acetonitrile (a weaker eluent) and isopropanol-ethyl acetate (a stronger eluent). The composition of the latter system, due to the great difference in the polarity of isopropanol and ethyl acetate, had the most pronounced effect on the analyte retention (Figure 2a). This is especially characteristic of amyrins, the most strongly retained compounds.

Since the highest concentrations of ethyl acetate led to an unacceptable deterioration in chromatographic resolution (*R_s_*) for some less retained analytes regardless of the composition of the eluent A, the value of 50% was chosen as the working content of this solvent in the eluent B. Furthermore, the use of a mixture of chemically different isopropanol and ethyl acetate facilitates the regeneration of the stationary phase at the end of the analytical cycle due to the effective removal of adsorbed matrix components of the analyzed sample, which is crucial in PGC-based chromatography. On the contrary, varying the ratio of methanol and acetonitrile in the eluent A (Figure 2b) had almost no effect on the retention of the PCTs. However, a 50% solution of acetonitrile in methanol provided the highest efficiency of the atmospheric pressure chemical ionization (APCI) and thus was chosen as the eluent A for further studies.

Surprisingly, the addition of formic acid to the mobile phase generally did not lead to noticeable changes in the retention of not only nonionic analytes, but also triterpenic acids (Figure 2c). This can be explained by the absence of water in the eluent and thus the suppression of their dissociation due to a decrease in the acidity constants of the carboxyl group. Despite this, the introduction of a minimal amount (0.01%) of formic acid resulted in an inexplicable decrease in the retention of all analytes by several percent. Likely, this effect is associated with the ability of HCOOH to act as an electronic surface modifier that reduces polar retention on the PGC. On the other hand, an increase in the acid concentration leads to a proportional increase in the efficiency of APCI in the positive ion mode, which justified the further use of 0.5% formic acid in the mobile phase composition. As in the case of formic acid, ammonium formate in the studied concentration range (0–10 mM) did not affect the PCTs retention (Figure 2d). This additionally confirms the absence of ionic interactions in the analyte-stationary phase system, the intensity of which is determined by the ionic strength of the medium.

As expected, an increase in column temperature led to a substantial decrease in analyte retention (Figure 2e). The observed pattern is quite typical for hydrophobic retention based on the dispersive interactions with the carbon surface. Higher temperatures are preferred in terms of analysis rapidity; however, they resulted in a loss of resolution between ursolic acid and 3*β*-taraxerol. In this regard, the temperature of 60 °C was chosen as preferable.

**Figure 3:**
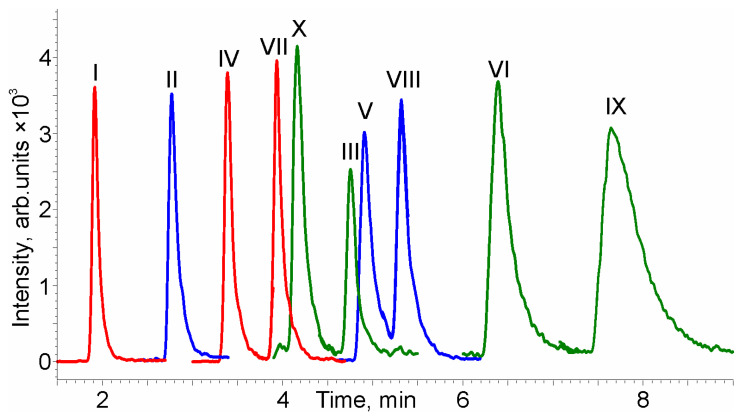
HPLC–MS/MS chromatogram of the analyte model mixture on the PGC stationary phase under the developed chromatographic conditions.

Thus, the rapid separation of the ten PCTs on the PGC stationary phase was achieved in 8 min (Figure 3, Table 1) using the gradient elution under the following conditions: eluent A—0.5% HCOOH in methanol-acetonitrile mixture (1:1, *v/v*); eluent B—0.5% HCOOH in isopropanol-ethyl acetate mixture (1:1, *v/v*); gradient program: 0 min—0% B, a linear ramp of B to 100% over 5 min, 5–10 min—100% B, equilibration to starting conditions for 5 min. The total duration of the analytical cycle is 15 min (including column flush and equilibration stages), which is 4–5 times less than that reported in the literature [30].

As can be seen from Figure 3, for most analytes, baseline separation was attained. The exceptions are two critical pairs of PCTs, ursolic acid—3*β*-taraxerol (VII–X) and lupeol-erythrodiol (III–V), for which *R_s_* values are 1.17 and 1.09, respectively. However, incomplete separation of these analytes does not cause problems for their identification and quantification, given that the components of these pairs are not isomers and are easily discriminated in mass spectrometric detection. Another drawback of the developed method is the typical PGC tailing of the peaks of highly retained analytes, primarily *β*- and *α*-amyrins, for which the asymmetry coefficients at 10% of the peak height (As_10%_) reach 3.1 and 3.9, respectively. This reduced the number of theoretical plates (N) obtained for *α*-amyrin to the level of 1700, while for some other analytes, N exceeded 10,000 (Table 1). The use of stronger eluents, as well as changes in ionic strength, column temperature, and the addition of formic acid, did not improve the peak shape of these analytes.

Special attention should be paid to the unusual elution order of the analytes closely associated with the specific retention mechanism. The retention factors (k) on PGC are not unambiguously determined by the belonging of analytes to one or another group in accordance with their polarity and chemical nature (monools, diols, and triterpenic acids). Thus, the diol betulin elutes in the group of triterpenic acids, while the remaining diols are characterized by retention times between those of the monools lupeol and *β*-amyrin. Changing the composition of the mobile phase did not affect the elution order of the analytes. This is in contrast with the earlier published data on the retention factors of PCTs in RP chromatography (triterpenic acids < diols < monools [4]), SFC on non-endcapped octadecyl stationary phases (triterpenic acids < monools < diols [27]), and mixed-mode HPLC on an Acclaim Mixed-Mode WAX-1 column (diols < monools < triterpenic acids [26]).

Non-polar compounds are retained on the PGC through dispersive interactions; thus, retention is determined by the hydrophobicity of the analyte, giving an elution order similar to that in an RP separation. However, the retention of the analytes bearing polar groups, lone pair electrons, or π-electrons of double bonds depends on their specific interactions with the polarizable PGC surface [34,35]. In this case, the geometry of the analyte’s molecule plays an important and, in some cases, decisive role. The flatter the molecule’s geometry, the closer it can approach the surface of the stationary phase, and thus the stronger the resulting interactions [28]. The contribution of such interactions to the retention mechanism explains the above-mentioned peculiarities in the elution order of PCTs. The presence of the available double C=C bond in the structure of PCTs increases their ability to be retained in the stationary phase. For example, the representatives of the ursane and oleanane families have a C=C bond in the structure of a six-membered ring between C-12 and C-13 carbon atoms and almost identical structures. The only difference between them lies in the position of the two methyl groups in the E cycle—in the oleanane-type structures, they are attached to the same C-20 carbon atom, while the oleanane family is distinguished by the methylated C-19 and C-20 carbon atoms. Therefore, ursane-type PCTs have a higher degree of planarity [36] and thus higher retention when compared with the similar compounds of the oleanane family. In the case of lupane-type compounds, the C-19 carbon atom has a prop-2-enyl substituent, which greatly distinguishes this family by having lower planarity and lower retention on PGC.

Another illustrative example of the retention specificity on the PGC is the separation of such extremely close in structure positional isomers as 3*β*-taraxerol (X) and *β*-amyrin (VI). Having the same polarity and differing only in the position of a double bond and a methyl group [37], these compounds cannot be effectively separated in an RP mode. At the same time, their retention on PGC differs dramatically—the measured k values for them were 10.3 and 16.2, respectively. An explanation of this phenomenon also involves the availability of the double bond for π-π interactions with the stationary phase. The C=C bond in *β*-amyrin is much more accessible since only one CH_3_ group is present at the C-14 atom. On the contrary, the double bond in 3*β*-taraxerol structure is located between C-14 and C-15 atoms, and adjacent to two methyl groups at C-13 and C-8 carbon atoms preventing its interaction with PGC. This factor leads to an abnormal position of the chromatographic peak of 3*β*-taraxerol between the peaks of much more polar triterpenic acids and diols.

### 2.2. Quantification and Method Validation

The instrumental limits of detection (LOD) and quantification (LOQ) of PCTs determined using 3σ and 10σ (signal-to-noise ratio) criteria, respectively, lie in the range of 4–20 (LOD) and 14–66 μg L^−1^ (LOQ) for the majority of the studied analytes (Table 2). An exception are the three most retained monools (uvaol, *α*- and *β*-amyrins) which have lower signal intensities due to the broadening of the chromatographic peaks and decreased APCI efficiency. The LODs for these analytes are 33, 61, and 104 μg L^−1^, respectively. Calibration dependences were linear (R^2^ > 0.99) in the range covering at least 2–3 orders of magnitude. The calculated LODs and LOQs were confirmed by recording an HPLC-MS/MS chromatogram of the analyte model mixture with concentrations close to the LOQ level (Supplementary Figure S1). The attained method sensitivity was at least two-fold higher compared to the GC-MS technique [19], even for *β*-amyrin and is close to that reported for the mixed-mode HPLC-MS/MS [26]. As expected, this observation does not apply to the three above-mentioned most retained analytes, for which the LOD values obtained in the present study turned out to be several times higher. In addition to the lower cost and wider availability of short Hypercarb columns compared to Acclaim Mixed-Mode WAX-1 [26], an important advantage of the proposed method is the chemical stability of the stationary phase, which allows the use of various solvents over the entire pH range as well as elevated temperatures for the regeneration of contaminated sorbent, which is extremely important when working with such complex matrices as plant extracts.

The results of the intra- and inter-day precision estimations showed that the developed method is characterized by good reproducibility and accuracy of quantitative determination. The standard deviation did not exceed 15%, and the accuracy was close to 100% (Supplementary Table S1). The matrix effects assessment was carried out by the spike recovery test at the three concentration levels using the birch xylem extract as a real matrix. The absence of matrix interferences in analyte quantification was proved by the high recovery values, which were in the range of 81–101% (Supplementary Table S2). The robustness of the developed method was confirmed by the slight variations of the column temperature (58–62 °C) and the eluent composition (within 1%), which did not lead to a substantial (>2%) shift in *t_R_* values and loss of chromatographic resolution *Rs*. An additional confirmation of the robustness is the analysis of dozens of plant extract samples with complex chemical compositions without deteriorating the separation parameters.

### 2.3. Plant Biomass Analysis

To test the developed method, the contents of PCTs in the herbaceous plant fireweed (leaves and stems) and two mosses—common haircap and prairie sphagnum—were determined. The choice of these plants was associated with their exceptional abundance in nature, their role in the formation of peat (mosses) [38,39,40], and poor knowledge of their triterpenoid composition, although these plants are considered a promising source of biologically active substances [41,42]. In the literature, there are no data on triterpenoids in the composition of secondary metabolites of common haircap, and fragmentary information on the detection of some PCTs in other objects of study is available [40,43] so far.

**Figure 4:**
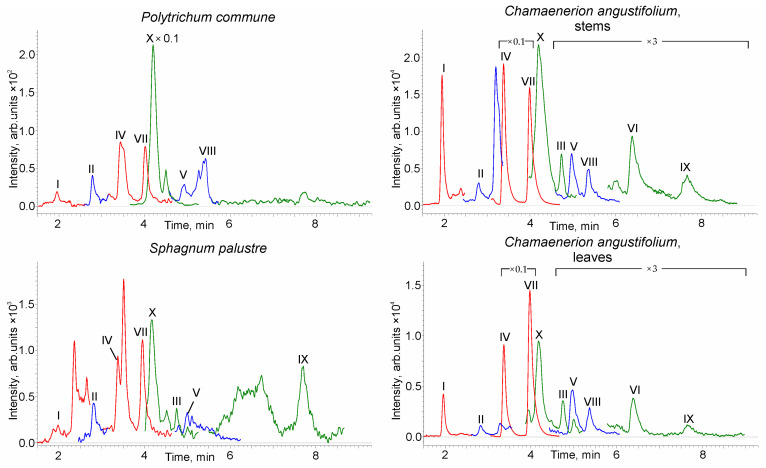
HPLC–MS/MS chromatograms of plant extracts.

The pressurized liquid extraction (PLE) of the PCTs from the plant biomass [44] was carried out by methanol, with the preliminary stage of the plant material cleanup by hot water extraction to remove excess polysaccharides contaminating the extracts. The obtained chromatograms (Figure 4) demonstrate the presence of all target analytes in the studied samples (the exceptions are the compounds III, VI, and IX in the haircap moss and VIII and VI in the prairie sphagnum samples). As can be seen, the most abundant representative of PCTs in common haircap moss is 3*β*-taraxerol, the content of which in the dry plant material was 0.18 mg g^−1^ (Table 3).

Other PCTs were found at levels of 0.68–12 μg g^−1^. The similar contents of PCTs (0.65–15 μg g^−1^) are also characteristic of another studied moss sample (prairie sphagnum). The major components detected in this plant were *α*-amyrin, ursolic acid, and 3*β*-taraxerol. It is worth noting that of all PCTs, only ursolic acid was previously reported in the literature [40] as a prairie sphagnum secondary metabolite. Fireweed is distinguished by the much higher (by two orders of magnitude) content of PCTs, most of which are concentrated in the leaves. The main components in both leaves and stems are oleanolic and ursolic acids, whose sum content in leaves reaches 0.6%. The leaf extract also contained a significant amount (800 μg g^−1^) of 3*β*-taraxerol. This significantly complements the literature data on the chemical composition of this plant, which reported only the detection of *α*- and *β*-amyrin, lupeol, uvaol, and erythrodiol [43].

Along with the signals of the target analytes, extraneous peaks of isomeric PCTs with the same MRM transitions are observed on the chromatograms. This is especially characteristic of the prairie sphagnum extract, on whose chromatogram the most intense peak with a retention time of 3.5 min remained unassigned. According to its MRM transition (*m/z* 409→95), this compound likely belongs to the class of monools. However, the reliable identification of such compounds requires their preparative isolation or the use of appropriate, scarcely available standards, which is beyond the scope of this study.

## 3. Materials and Methods

### 3.1. Reagents and Materials

Ten commercially available pentacyclic triterpenoids—betulinic acid (≥97.0%), oleanolic acid (≥97.0%), ursolic acid (≥90.0%), lupeol (≥90.0%), *α*-amyrin (≥98.5%), *β*-amyrin (≥98.5%), 3*β*-taraxerol (≥95%), betulin (≥98.0%), erythrodiol (≥97.0%), and uvaol (≥95.0%) were purchased from Sigma-Aldrich (Steinheim, Germany).

HPLC gradient grade methanol, acetonitrile, ethyl acetate, isopropanol (Chimmed, Moscow, Russia), formic acid (≥96%, Sigma-Aldrich, St. Louis, MO, USA), and ammonium formate (10 M aqueous solution, Sigma-Aldrich, St. Louis, MO, USA) were used for the preparation of the mobile phase and in the plant material pressurized liquid extraction (PLE) procedure. Deionized water with a resistivity of 18.2 MΩ cm was obtained using a Simplicity UV system (Millipore, Molsheim, France).

The stock solutions of PCTs in methanol (250 mg L^−1^) were prepared from an accurately weighed sample. Calibration solutions of analytes were obtained by mixing successive dilutions of the stock solutions with methanol. All prepared solutions were stored in the dark at 4 °C for no more than one week. Their stability was checked once a day.

### 3.2. Plant Materials and Extraction Procedure

Common haircap (*Polytrichum commune*) and prairie sphagnum (*Sphagnum palustre*), mosses, fireweed (*Chamaenerion angustifolium*) stems and leaves, and birch xylem (*Betula pendula*)—were harvested in the forests of the Arkhangelsk region of Russia in August 2022. The identification of the plant material’s botanical origin was carried out according to the herbarium of the Northern (Arctic) Federal University.

The accelerated solvent extraction system ASE-350 (Dionex, Sunnyvale, CA, USA) was used for pressurized liquid extraction of plants. The samples of dry plant material (1.0 g) were extracted with methanol (two extraction cycles of 10 min each) at 100 °C and 100 bar under a nitrogen atmosphere with preliminary water extraction in two cycles. The resulting extracts were dried and dissolved (1 mg mL^−1^) in methanol, then diluted to the final concentration, filtered through a nylon membrane filter (0.22 μm), and subjected to chromatographic analysis. The birch xylem PLE extract was used for the estimation of matrix effects.

### 3.3. Liquid Chromatography—Mass Spectrometry Analysis

The chromatographic analysis was carried out on an LCMS-8040 high-performance liquid chromatography—tandem mass spectrometry system (Shimadzu, Kyoto, Japan), consisting of a Nexera LC-30 liquid chromatograph and triple quadrupole mass analyzer. An HPLC system consisted of a DGU-5A vacuum degasser, an LC-20AD chromatographic pump, an SIL-20AC autosampler, and a CTO-20AC column thermostat. The chromatographic separation was carried out on a Hypercarb column, 30 × 3.0 mm, 3.0 µm particle size (Thermo Scientific, Waltham, MA, USA), with the porous graphitic carbon stationary phase at 60 °C. The mobile phase consisted of eluent A (methanol-acetonitrile, 1:1) and eluent B (ethyl acetate—isopropanol, 1:1), both containing 0.5% formic acid. The following gradient program was used: 0 min—0% B; 5 min—100% B; 5–10 min—100% B; equilibration to starting conditions for 5 min. The flow rate was 0.8 mL min^−1^. Injection volume was 10 μL. The system control and data treatment were performed using LabSolutions 5.56 software (Shimadzu, Kyoto, Japan).

The mass spectrometry detection was carried out in atmospheric pressure chemical ionization (APCI) mode with the following ion source parameters optimized in the preliminary experiments: interface temperature—350 °C; desolvation line temperature—250 °C; heat block temperature—250 °C; nebulizing gas flow rate—4 L min^−1^; drying gas flow rate—15 L min^−1^. Since PCTs easily undergo in-source fragmentation with the loss of a water molecule [4], the corresponding protonated dehydrated analyte molecules [M − H_2_O + H]^+^ were chosen as precursor ions. Their list, as well as the optimized parameters of MPM transitions, are presented in Table 4.

### 3.4. Method Validation

The limits of detection (LOD) and quantification (LOQ) were determined using the signal-to-noise ratio (S/N) criteria of 3 and 10, respectively, and then refined in the analysis of a model analyte solution with concentrations close to LOQ. The estimation of the intra-day precision was determined by consecutive chromatographic analyses (*n* = 7) of the analyte model mixture at the lowest (close to LOQ) concentration level. An inter-day precision was determined in the same way within 48 h (*n* = 14). To estimate the matrix effect and accuracy of quantification, the spike-recovery test was used. The analytes at three concentration levels were added to a PLE extract of birch xylem and then analyzed in triplicate.

## 4. Conclusions

Due to the combination of hydrophobic retention and specific interactions between polar groups or π-electrons of analytes with the polarizable graphite surface, porous graphitic carbon stationary phase (Hypercarb) is capable of the rapid and efficient chromatographic separation of pentacyclic triterpenoids of various classes, including structurally close positional isomers. The study of the effects of the mobile phase composition, pH, ionic strength, and column temperature on the retention of ten PCTs on Hypercarb allowed for the development of the chromatographic conditions and separation of triterpenic acids, diols, and monools within 8 min in gradient elution mode. On this basis, a porous graphitic carbon HPLC-MS/MS method for the simultaneous determination of betulinic, oleanolic, and ursolic acids, lupeol, *α*- and *β*-amyrins, 3*β*-taraxerol, betulin, erythrodiol, and uvaol in plant extracts was developed and fully validated. The attained limits of detection lie in the range of 4–104 µg L^−1^ and are comparable with those reported in the literature for the HPLC-MS methods with the reversed phase and mixed-mode separation techniques. The advantages of the developed method are its rapidity, low cost, and chemical stability in the stationary phase. The combination of the developed method with pressurized liquid extraction as a sample preparation technique was successfully used for the determination of the PCTs in common haircap (*Polytrichum commune*) and prairie sphagnum (*Sphagnum palustre*) mosses and fireweed (*Chamaenerion angustifolium*) stems and leaves. It was found that 3*β*-taraxerol and *α*-amyrin predominate in the moss biomass, while fireweed leaves are distinguished by their high content (0.6%) of oleanolic and ursolic acids.

## Figures and Tables

**Table 1:** Chromatographic separation parameters * of the analytes under the developed chromatographic conditions.

Analyte	t_R_	k	α	N	*R_s_*	As_10%_	As_50%_
I	1.93	4.2	-	3750	-	2.12	1.15
II	2.81	6.6	1.56	4410	5.98	2.81	1.30
III	4.74	11.8	1.15	11,900	2.91	2.68	1.49
IV	3.38	8.1	1.23	8030	3.57	2.09	1.24
V	4.96	12.4	1.05	7330	1.09	2.92	1.69
VI	6.38	16.2	1.20	5130	3.59	3.05	1.46
VII	3.97	9.7	1.20	11,400	3.95	2.84	1.37
VIII	5.36	13.5	1.09	10,100	1.80	2.76	1.23
IX	7.69	19.8	1.22	1730	2.40	3.86	2.62
X	4.18	10.3	1.06	6210	1.17	2.89	1.53

* t_R_—retention time; k—retention factor; α—selectivity; N—theoretical plate number; *R_s_*—chromatographic resolution; A_s10%_ and A_s50%_—asymmetry coefficients at 10% and 50% peak height, respectively.

**Table 2:** Calibration dependences of the chromatographic peak areas on the concentrations of the analytes (*y = ax*), limits of detection, and quantification of ten PCTs.

Analyte	t_R_, min	Linear Range, μg L^−1^	a	R^2^	LOD, μg L^−1^	LOQ, μg L^−1^
I	1.93	16–5000	74.9	0.999	4.69	15.6
II	2.81	39–10,000	52.2	0.999	11.7	38.9
III	4.74	23–5000	78.4	0.994	6.85	22.8
IV	3.38	14–5000	100	0.999	4.26	14.2
V	4.96	66–15,000	35.9	0.999	19.9	66.3
VI	6.38	200–40,000	28.8	0.999	60.6	202
VII	3.97	34–10,000	50.5	0.999	10.2	34.1
VIII	5.36	110–25,000	24.2	0.999	32.8	109
IX	7.69	350–70,000	29.0	0.999	104	347
X	4.18	34–10,000	64.8	0.999	10.0	33.5

**Table 3:** The content of PCTs (μg g^−1^, recalculated for the oven-dried sample) in plant materials.

Analyte	Common Haircap	Prairie Sphagnum	Fireweed Stems	Fireweed Leaves
I	0.68 ± 0.13	0.65 ± 0.03	49 ± 4	87 ± 5
II	1.8 ± 0.1	2.7 ± 0.3	15 ± 1	32 ± 1
III	<LOQ	1.6 ± 0.2	4.7 ± 0.3	21 ± 1
IV	2.5 ± 0.2	3.0 ± 0.1	550 ± 30	1500 ± 100
V	3.6 ± 1.5	3.6 ± 0.3	21 ± 1	110 ± 10
VI	<LOQ	<LOQ	48 ± 2	140 ± 10
VII	4.3 ± 0.3	8.4 ± 0.1	840 ± 10	4500 ± 100
VIII	12 ± 1	<LOQ	22 ± 1	90 ± 4
IX	<LOQ	15 ± 2	32 ± 7	79 ± 6
X	180 ± 1	8.0 ± 0.3	210 ± 10	800 ± 10

**Table 4:** Optimized conditions for PCT mass spectrometric detection in MRM mode.

Analyte	Nominal Mass, Da	Precursor Ion, [M − H_2_O + H]^+^ *m*/*z*	Product Ion, *m*/*z*	Q1 Bias, V	Collision Energy, eV	Q2 Bias, V
I	456	439	95	−46.8	40	−40.3
II	442	425	95	−43.5	32	−37.1
III	426	409	95	−43.5	33	−14.5
IV	456	439	203	−50.0	27	−43.5
V	442	425	191	−46.8	14	−46.8
VI	426	409	95	−43.5	36	−43.5
VII	456	439	203	−46.8	26	−40.3
VIII	442	425	191	−46.8	17	−37.1
IX	426	409	95	−40.3	40	−37.1
X	426	409	95	−10.3	38	−36.1

## Data Availability

The data presented in this study are available in the article and Supplementary Materials.

## Supplementary Materials

The following supporting information can be downloaded at: https://www.mdpi.com/article/10.3390/molecules28093945/s1. Table S1: Intra-day and inter-day precision of the PCT determination by porous graphitic carbon HPLC-MS/MS. Table S2: Matrix effect on the determination of PCTs by porous graphitic carbon HPLC-MS/MS estimated in the spike recovery test. Figure S1: HPLC–MS/MS chromatogram of analytes model mixture with the concentrations close to LOQ.
