# Supplementary material for: Determination of Pentacyclic Triterpenoids in Plant Biomass by Porous Graphitic Carbon Liquid Chromatography—Tandem Mass Spectrometry
Source: Molecules. 2023 May 7;28(9):3945. doi: 10.3390/molecules28093945 (PMC10180310; doi:10.3390/molecules28093945)
Supplement: Supplementary file 1 [file molecules-28-03945-s001.zip › molecules-2380783-supplementary.pdf]

Supplementary Material

Table S1. Intra-day and inter-day precision of the of the PCTs determination by porous graphitic carbon HPLC-MS/MS

Analyte	Concentration, μg L^{-1}	Intra-day assay			Inter-day assay		
		Measured concentration μg L^{-1}	Accuracy, %	Precision, %	Measured concentration μg L^{-1}	Accuracy, %	Precision, %
I	25	28.4±1.3	114	5.17	24.2±0.9	96.9	5.09
II	50	48.1±1.4	96.2	3.32	49.1±2.5	98.3	6.86
IV	25	25.4±1.2	101	5.44	23.8±0.9	95.4	4.84
VII	50	49.5±2.4	99	5.62	45.6±7.1	91.3	9.54
X	50	53.7±4.1	107	8.57	54.4±3.8	105	9.84
III	25	23.5±2.1	94.2	9.25	24.9±3.1	99.7	14.4
V	75	87.6±7.0	117	7.95	77.7±2.2	104	3.75
VIII	125	124±12	99.5	11.2	126±7	101	6.71
VI	200	217±13	109	6.85	206±2	103	1.31
IX	350	315±26	89.9	9.61	406±14	116	4.03

Table S2. Matrix effect on the determination of PCTs by porous graphitic carbon HPLC-MS/MS estimated in the spike recovery test.

**Analyte**	**Initial concentration, μg L⁻¹**	**Spiked, μg L⁻¹**	**Found, μg L⁻¹**	**Recovery, %**
I	5.55±1.54	25	27.8±2.2	91
		250	210±3	82
		500	466±11	92
II	66.3±5.2	50	118±3	101
		500	527±3	93
		1000	1035±10	97
IV	1.82±0.73	25	23.8±3.3	89
		250	210±5	84
		500	476±7	95
VII	–	50	42.5±2.5	85
		500	424±23	85
		1000	974±18	97
X	–	50	40.7±2.7	81
		500	449±40	90
		1000	936±12	94
III	11.3±2.3	25	34.4±2.1	95
		250	246±7	94
		500	452±7	88
V	–	75	67.5±2.8	90
		750	651±30	87
		1500	1438±13	96
VIII	–	125	123±11	99
		1250	1102±25	88
		2500	2225±16	89
VI	–	200	187±4	93
		2000	1713±12	86
		4000	3820±119	96
IX	–	350	289±32	83
		3500	3210±39	92
		7000	6670±31	95

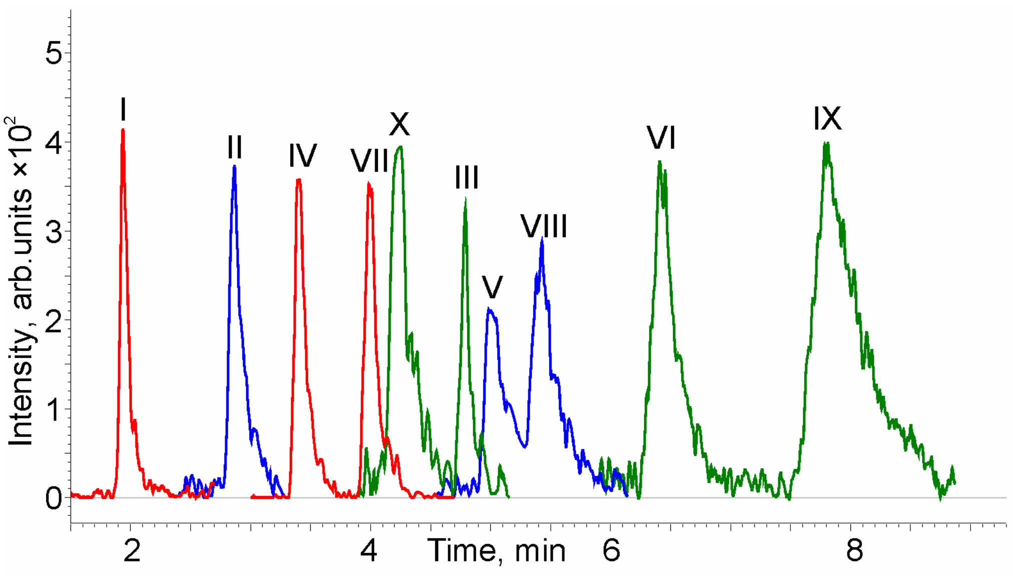

Figure S1. HPLC–MS/MS chromatogram of analytes model mixture with the concentrations close to LOQ (I, IV and III - 25 μg L^{-1}; II, VII and X – 50 μg L^{-1}; V – 75 μg L^{-1}; VIII - 125 μg L^{-1}; VI – 200 μg L^{-1}; IX – 350 μg L^{-1}).
